# Cavitating Mesenteric Lymph Node Syndrome With Autoimmune Pancreatitis and Hepatitis: A Case Report and Literature Review

**DOI:** 10.7759/cureus.90183

**Published:** 2025-08-15

**Authors:** Aoumar Chama, Wendy Saliba, Anthony Nasr

**Affiliations:** 1 Cardiology, University of Balamand, Beirut, LBN; 2 Radiology, Lebanese University, Beirut, LBN

**Keywords:** autoimmune hepatitis, autoimmune pancreatitis (aip), cavitating mesenteric lymph node syndrome (cmlns), celiac disease and autoimmunity, systemic autoimmune disease

## Abstract

Cavitating mesenteric lymph node syndrome (CMLNS) is a rare and severe complication of refractory celiac disease. It is characterized by the triad of cavitating mesenteric lymph nodes, splenic atrophy and villous atrophy of the small bowel mucosa.

We present the case of a 36-year-old male patient with a known diagnosis of celiac disease who presented with fever and diarrhea. Further evaluation revealed progression to refractory celiac disease and he developed the rare complication of CMLNS along with concomitant autoimmune hepatitis and autoimmune pancreatitis. We concluded from our case that despite adherence to a strict gluten-free diet and high-dose corticosteroids, cavitary mesenteric lymph node syndrome is highly fatal and leads to complications like septic shock and gastrointestinal perforation.

We emphasize the critical need for early recognition of refractory celiac disease and highlight the potential of associated autoimmune disorders, which significantly complicate patient management. Timely diagnosis of complications such as autoimmune pancreatitis and hepatitis is crucial, as they necessitate distinct therapeutic approaches. Our case underlines the urgency of developing more effective treatment strategies to improve patient outcomes.

## Introduction

Celiac disease (CD) is defined as an autoimmune disorder whereby the ingestion of gluten (e.g., wheat, barley) triggers an immune response that damages the intestinal lining. It presents as abdominal distention, diarrhea, vomiting, anorexia and weight loss [1]. Also, some extraintestinal manifestations can be present like anemia, short stature and neurological manifestations [2].

CD could be associated with other autoimmune diseases like chronic active hepatitis in some cases, type I diabetes, and autoimmune thyroid disease [3]. Also, it has been linked to an increased risk of both acute and chronic pancreatitis [4-6]. Chronic inflammation in CD can compromise mucosal immunity and systemic immune regulation, contributing to multi-organ involvement. One of the rare complications of refractory celiac disease (RCD) is cavitating mesenteric lymph node syndrome (CMLNS), which is associated with a bad prognosis (50% mortality rate) due to many complications such as severe malnutrition, intestinal ulceration that leads to hemorrhage, sepsis due to hyposplenism and reduced IgM memory B-cells [7] as well as lymphoma, most commonly enteropathy-associated T-cell lymphoma (EATL) [8,9].

In this report, we describe a rare case of CMLNS in a patient with RCD, complicated by autoimmune hepatitis (AIH) and autoimmune pancreatitis (AIP). This case underscores the importance of early recognition of atypical disease presentations and the need for multidisciplinary management in patients with complex autoimmune gastrointestinal disorders.

## Case presentation

A 36-year-old man, known to have CD (diagnosed seven months ago by intestinal biopsy), presented to the emergency room for fever and diarrhea for the past four weeks.

The patient described his diarrhea as being profuse, watery, foul-smelling and non-bloody. It is associated with abdominal pain and weight loss despite maintaining a good appetite.

On examination, the patient appeared icteric and cachectic but was conscious, oriented, and hemodynamically stable. Mild hepatomegaly was notable on abdominal palpation.

On laboratory exam, serum lipase was normal (44 IU). His liver function test revealed a predominantly direct hyperbilirubinemia (total bilirubin of 32 mmol/l with a direct bilirubin of 28 mmol/l), elevated serum alanine transaminases (alanine aminotransferase (ALT) of 566 U/l, aspartate aminotransferase (AST) of 478 IU/l) and serum alkaline phosphatase of 181 U/l. His serum albumin level was 2.5 g/dl. Viral hepatitis serology (hepatitis B surface antigen (HBsAg), anti-hepatitis C virus (HCV), IgM hepatitis E virus (HEV), IgM hepatitis A virus (HAV)) and antimitochondrial antibodies were negative. However, autoimmune markers were positive: antinuclear antibody (ANA) titer of 1:100 and anti-smooth muscle antibodies. Serum IgG4 levels were significantly elevated at 2.3 g/L (reference range: 0.04-0.87). Anti-tissue transglutaminase IgA levels were >200 IU/mL (reference: <20 IU/mL).

**Figure 1:**
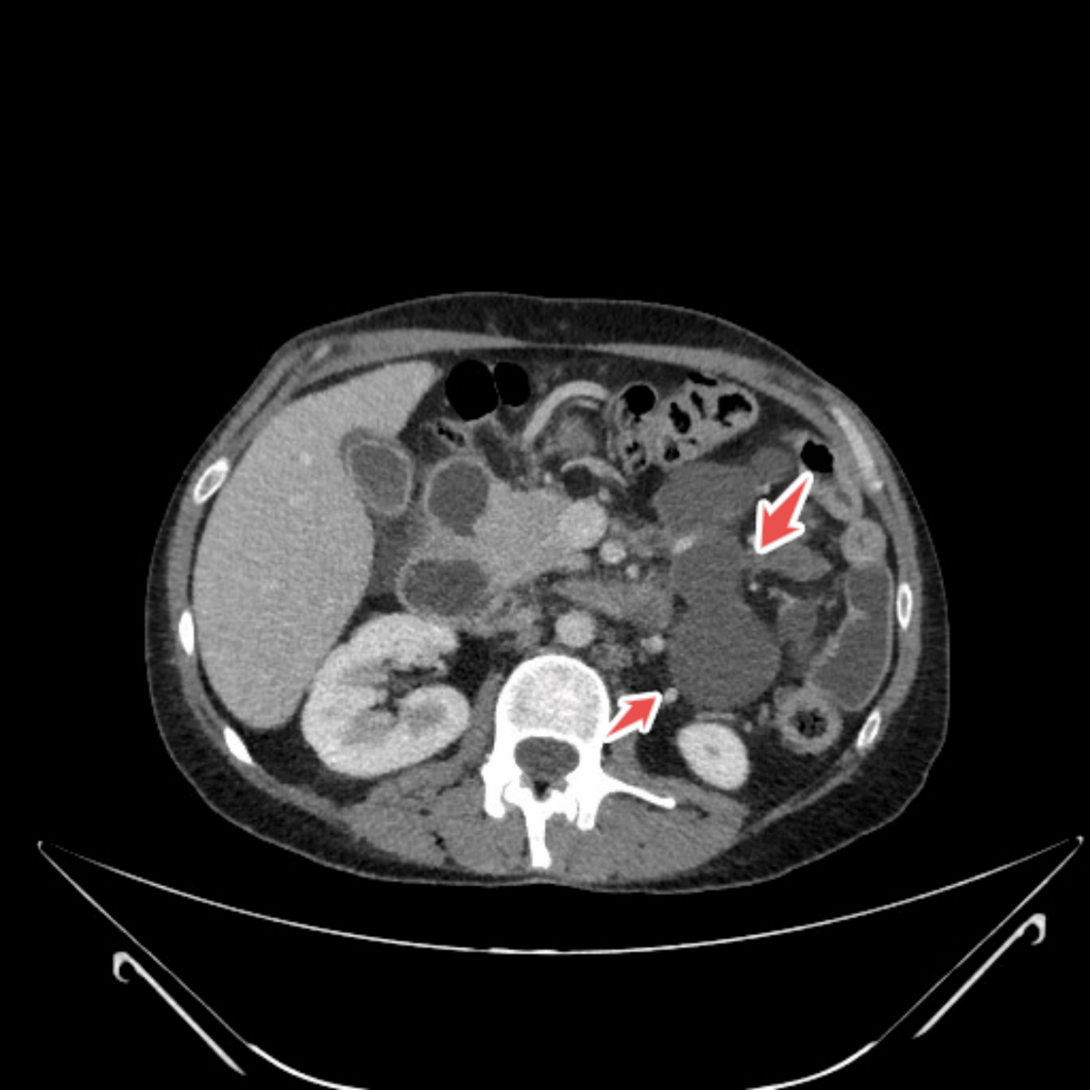
CT abdomino-pelvic showing loculated cystic structures in the left upper quadrant highlighted by arrows

**Figure 2:**
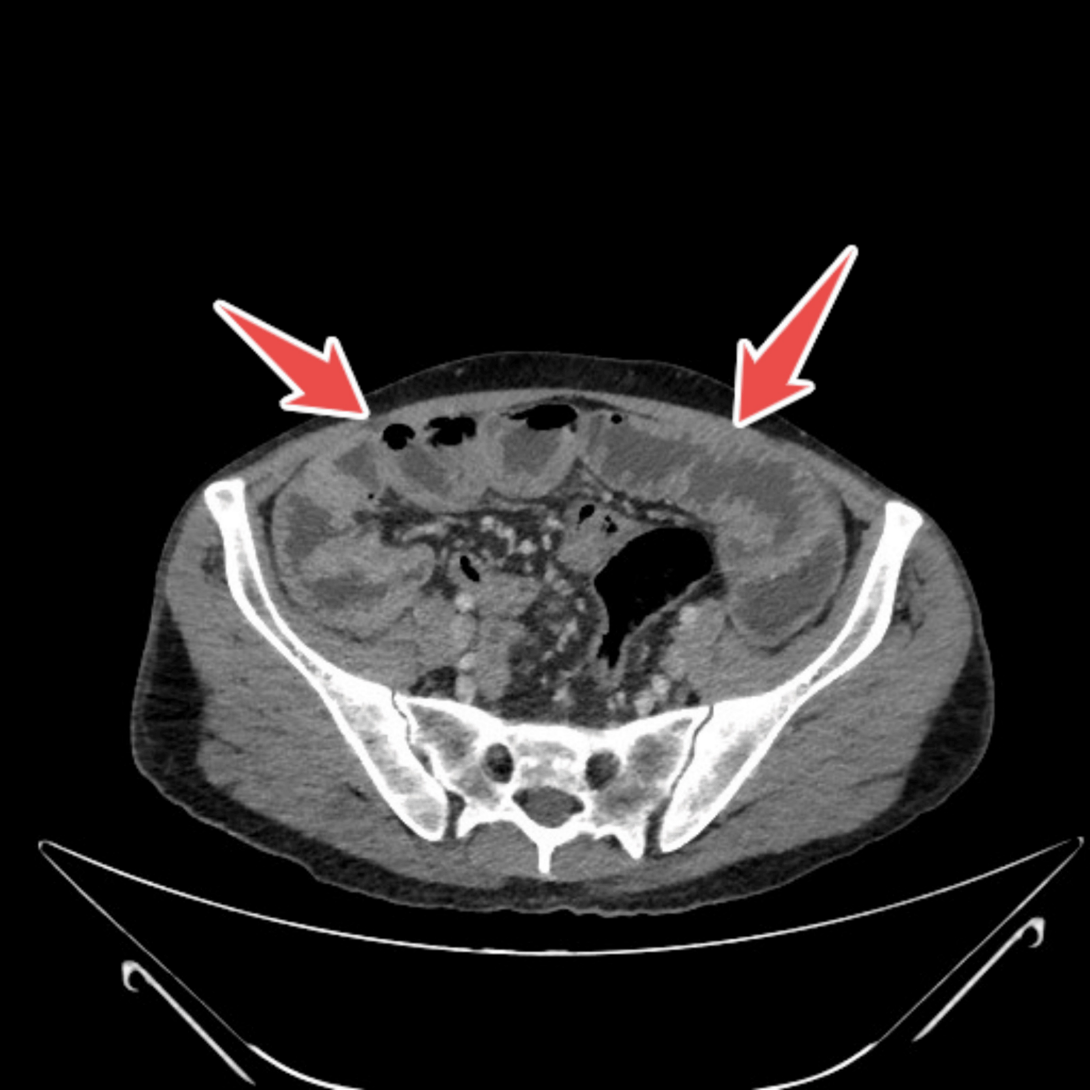
CT abdomino-pelvic showing relative absence of jejunal folds, thickening of the ileum, up to 1 cm in some areas highlighted by arrows

**Figure 3:**
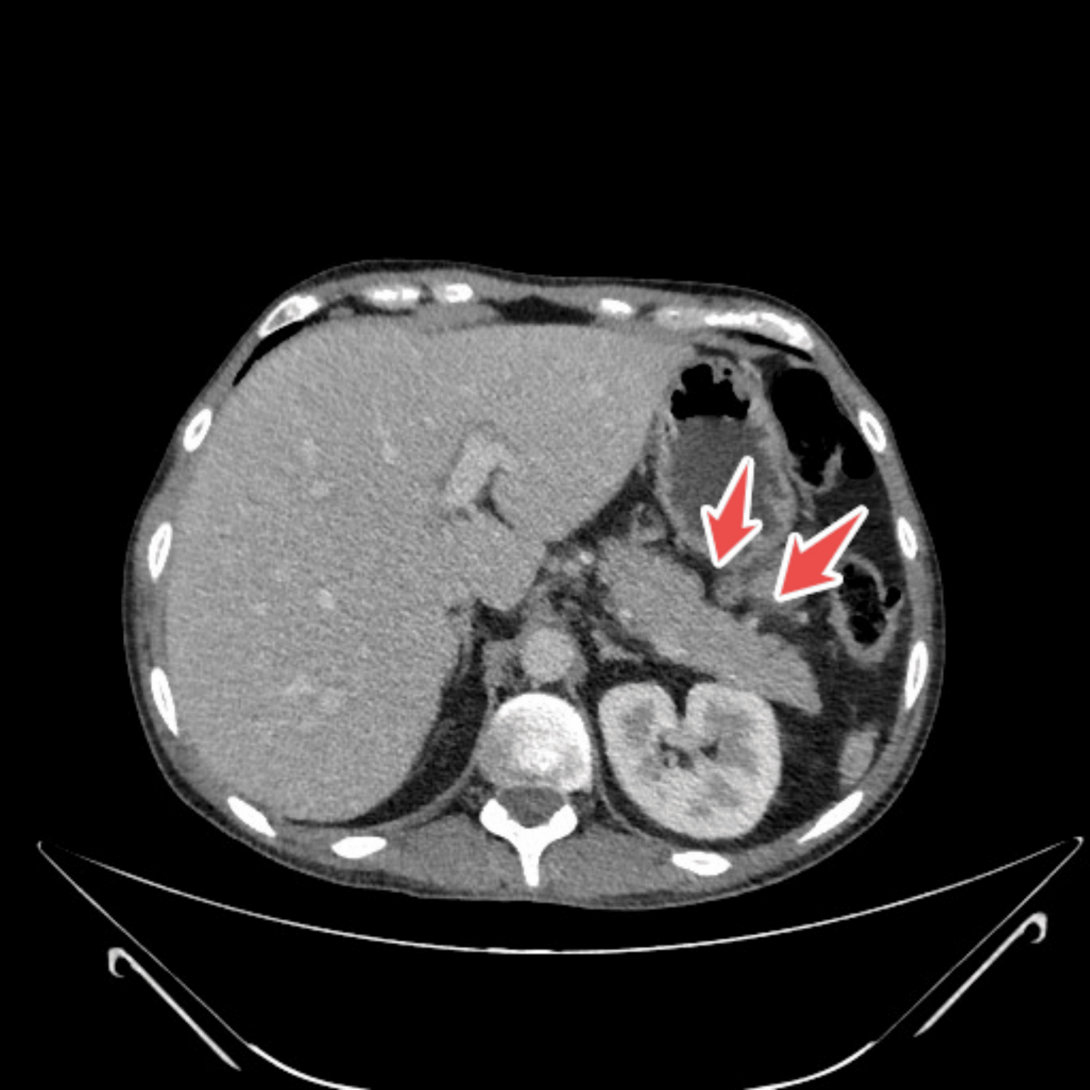
Ct abdomino-pelvic showing spleen atrophy highlighted by arrows

CT scan of the thorax, abdomen and pelvis revealed prominent hilar and mediastinal nodes, mild prominent mesenteric and retroperitoneal nodes (largest one being 10 mm) and some loculated cystic structures in the left upper quadrant (largest 8x5 cm), an enlarged pancreas, a mildly enlarged liver, atrophy of spleen, the relative absence of jejunal folds, thickening of the ileum up to 1 cm in some areas, with markedly increase folds and the presence of mesenteric hypervascularity (Figure 1, Figure 2, Figure 3).

Based on imaging and clinical history, a diagnosis of CMLNS was established. Autoimmune hepatitis and autoimmune pancreatitis were also suspected. Thus, the patient was started on a strict gluten-free diet and took high-dose steroid therapy following a broad-spectrum antibiotic treatment (piperacillin-tazobactam 4.5g IV every six hours).

Ultrasound-guided fine needle aspiration (FNA) of a mesenteric cyst revealed pleomorphic and polymorphic lymphocytosis with atypical large cells. An excisional biopsy of an accessible peripheral lymph node was recommended for histologic confirmation.

A transjugular liver biopsy showed features consistent with autoimmune hepatitis with advanced fibrosis and early cirrhosis. Cultures grew methicillin-sensitive Staphylococcus aureus (MSSA). Accordingly, antibiotic therapy was de-escalated to intravenous cefazolin (2g IV every six hours) as targeted therapy.

**Figure 4:**
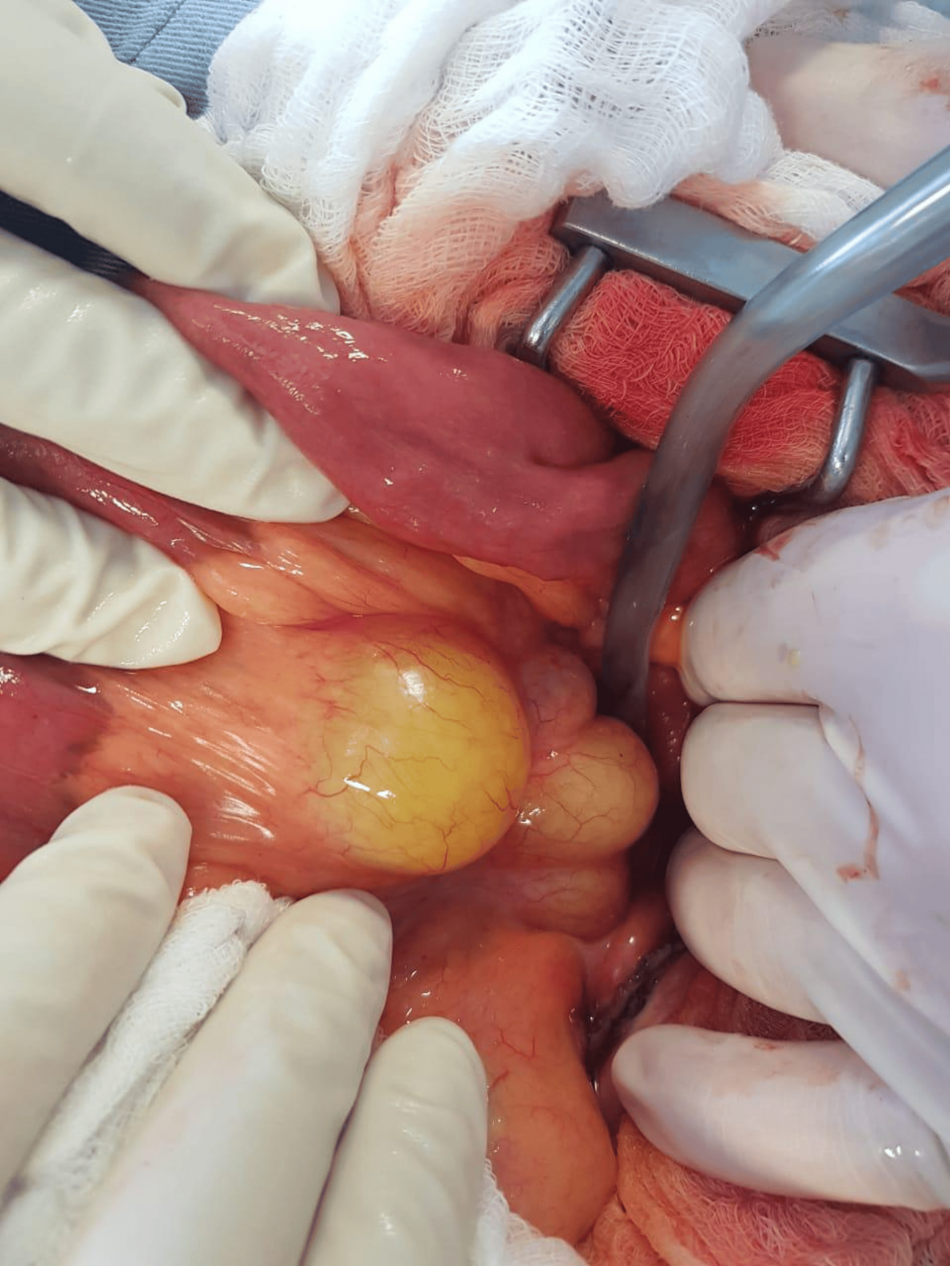
Mesenteric cysts in abdomen

Despite these therapies, the patient had a persistent high-grade fever (39-40 degrees) associated with abdominal pain. Therefore, intestinal lymphoma had to be ruled out. Exploratory laparotomy with mesenteric cyst excisional biopsy and fluid cyst aspiration was done (Figure 4).

Immunohistochemistry of the cyst biopsy from the left hypochondrium revealed CD3-positive T cells and CD20-positive B cells, consistent with non-specific inflammatory serosal cysts, without evidence of neoplasia. Cytological analysis of the cyst fluid showed lymphocytosis.

**Figure 5:**
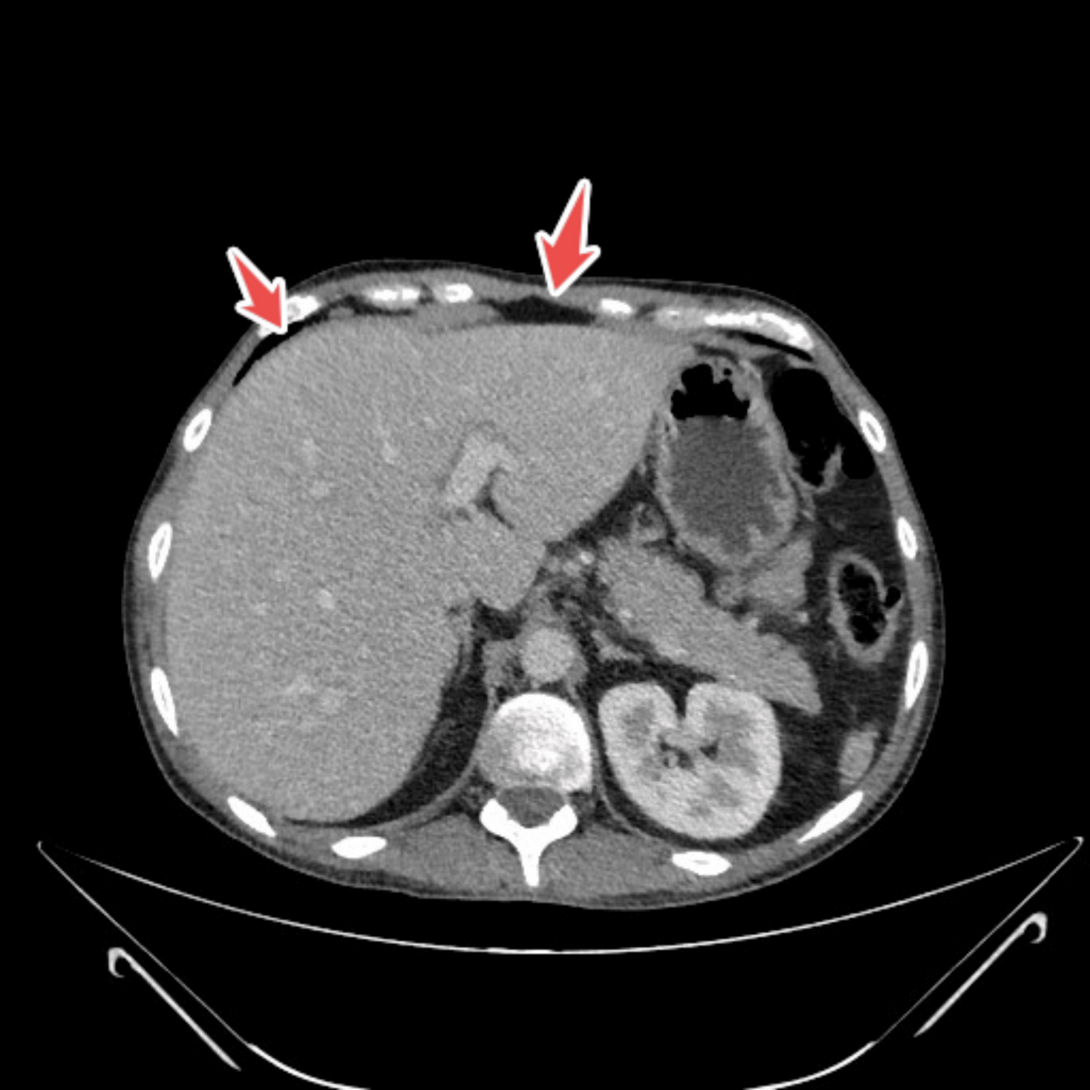
CT abdomino-pelvic showing pneumoperitoneum, highlighted by arrows.

Day 12 post-laparotomy, our patient started developing severe abdominal pain in association with fever and watery diarrhea. C-diff (Clostridioides difficile) testing was negative. Physical exam of the abdomen showed rigidity with guarding; therefore urgent CT abdominopelvic was done and showed pneumoperitoneum due to suspected gastroduodenal perforation with abdominal ascites (Figure 5).

After that, the patient was transferred to the intensive care unit (ICU) and empiric antibiotic therapy was escalated to intravenous meropenem (1g every eight hours) and vancomycin, to provide broad-spectrum coverage including anaerobes, gram-negative bacilli, and methicillin-resistant Staphylococcus aureus (MRSA), in light of his immunocompromised status and prior MSSA infection. A nasogastric tube was inserted, and abdominal paracentesis was performed.

The patient started being confused on day three post-ICU transfer due to the development of hepatic encephalopathy and he was managed with lactulose (30 ml every three hours). He subsequently developed hematochezia with hemoglobin drop (6.8 g/dL), so blood transfusion was started. CT imaging was repeated and showed colitis and bilateral pleural effusions.

On day five post-transfer, the patient became hypoxic, hypotensive, and tachycardic (clinical signs consistent with septic shock). Therefore, he was intubated, sedated with midazolam, and started on norepinephrine to maintain mean arterial pressure.

Despite aggressive supportive measures, the patient continued to experience profuse hematochezia, so an urgent upper endoscopy (gastroscopy) was done and showed a normal esophagus and gastric mucosa, with no identifiable source of upper gastrointestinal bleeding.

On ICU day six, the patient suffered three consecutive episodes of cardiopulmonary arrest, each preceded by marked bradycardia and hypotension. Advanced cardiac life support (ACLS) was initiated; however, return of spontaneous circulation (ROSC) was not achieved, and the patient was declared deceased.

## Discussion

CD represents a chronic autoimmune enteropathy that develops in genetically susceptible individuals after consumption of gluten. The immune system causes damage to the small intestinal mucosa, which results in malabsorption and multiple gastrointestinal and extraintestinal symptoms. The condition has been linked to multiple autoimmune disorders, demonstrating its widespread immunological effects on the body [1,2].

In rare and severe cases, CD may evolve into RCD, defined by the persistence or recurrence of malabsorptive symptoms and villous atrophy despite strict adherence to a gluten-free diet for at least six to 12 months [8]. The development of RCD leads to an elevated risk of developing malignancy, especially EATL, and increases the chance of severe complications including CMLNS [8,10,11].

CMLNS represents a rare and dangerous condition that develops from RCD complications. The condition presents with three main features which include cavitating mesenteric lymph nodes together with splenic atrophy that leads to hyposplenism and persistent small bowel villous atrophy [12]. The pathogenesis of this condition involves two possible mechanisms: hemorrhagic infarction from chronic immune stimulation or lymphatic obstruction with chyle accumulation which may lead to lymph node cavitation [12].

Our patient displayed typical radiologic signs of CMLNS through multiple mesenteric cystic lesions (up to 8 × 5 cm), splenic atrophy, and jejunal fold loss with ileal thickening [13,14]. The pathognomonic features for CMLNS in CD patients include fat-fluid levels inside cavitating nodes [13].

The clinical course of our patient was further complicated by AIH and AIP. The combination of elevated transaminases with positive autoimmune markers (ANA, anti-smooth muscle antibodies) and elevated IgG4 levels and liver biopsy results confirmed AIH while imaging results and elevated IgG4 levels indicated AIP Type 1. CD has been linked to rare instances of autoimmune hepatic and pancreatic disorders according to previous research [3-5,15]. The pathophysiology may involve increased intestinal permeability, allowing translocation of immune complexes or anti-tTG antibodies cross-reacting with extraintestinal tissues [15].

CMLNS patients commonly develop splenic atrophy which weakens their immune system and makes them more vulnerable to infections from encapsulated bacteria like Streptococcus pneumoniae and Haemophilus influenzae [7]. This likely played a role in the development of MSSA sepsis in our patient, despite broad-spectrum antibiotic therapy.

Additionally, our patient developed hematological complications which included thrombocytopenia together with coagulopathy. CD patients rarely experience these complications which stem from either autoimmune platelet destruction or vitamin K malabsorption caused by villous atrophy [16,17].

Despite initial improvement with corticosteroids and gluten withdrawal, the patient’s condition deteriorated, culminating in intestinal perforation, sepsis, hepatic encephalopathy, and ultimately fatal multiorgan failure. The absence of identifiable bleeding on upper endoscopy and the presence of colitis and pleural effusions on imaging further complicated his management.

This case illustrates the diagnostic and therapeutic challenges posed by rare systemic complications of CD. It emphasizes the importance of a multidisciplinary approach, incorporating gastroenterology, hepatology, immunology, and intensive care teams, particularly when managing patients with refractory disease and suspected autoimmune overlap syndromes.

## Conclusions

The case presents an unusual severe clinical presentation of RCD that included CMLNS alongside AIH and AIP. Despite adherence to a strict gluten-free diet and initiation of high-dose corticosteroids, the patient’s condition rapidly deteriorated, culminating in multiorgan involvement, gastrointestinal perforation, septic shock, and death.

The coexistence of CMLNS with autoimmune hepatic and pancreatic involvement highlights the complex systemic nature of advanced CD and the need for early recognition of atypical and extraintestinal manifestations. The presence of splenic atrophy, immune dysfunction, and severe gastrointestinal complications underscores the importance of timely imaging, histologic confirmation, and aggressive multidisciplinary management

This case demonstrates the immediate requirement for improved diagnostic strategies and targeted immunotherapeutic approaches for patients who have RCD and systemic autoimmune conditions. Additionally, it reinforces the value of close clinical monitoring and collaborative care involving gastroenterologists, hepatologists, immunologists, and intensivists in managing such complex and rapidly evolving presentations.
